# Effect of high sodium ion level on the interaction of AmB with a cholesterol-rich phospholipid monolayer

**DOI:** 10.3389/fmolb.2024.1405383

**Published:** 2024-05-09

**Authors:** Juan Wang, Jiahao Qiang, Jinzi Li, Dengwu Wang

**Affiliations:** ^1^ Xi’an Key Laboratory of Advanced Photo-electronics Materials and Energy Conversion Device, School of Electronic Information, Xijing University, Xi’an, China; ^2^ Shaanxi Engineering Research Center of Controllable Neutron Source, School of Electronic Information, Xijing University, Xi’an, China

**Keywords:** monolayer, amphotericin B, atomic force microscopy, sodium ions, cholesterol

## Abstract

Invasive fungal infections are a primary reason for high mortality in immunocompromised people, especially in critically ill patients, such as intensive care unit (ICU) patients, advanced cancer patients, or severe burn patients. Hypernatremia also can increase mortality in severely ill patients. Amphotericin B (AmB) is the gold standard for treating infections, but in severely ill patients, AmB can cause hematotoxicity when administered intravenously due to its interaction with cholesterol on red blood cell membranes. This results in limited doses of AmB and affects the treatment of infections. The proportion of cholesterol molecules in membrane lipids in red blood cells is as high as 50 mol%, and the sodium ions can influence the interaction between AmB and lipids on the membrane. Therefore, in the complex clinical situation of a severely ill patient with a fungal infection and hypernatremia, the interaction between amphotericin B and the red blood cell membranes is worth studying in depth. In this work, the interaction between AmB and the dipalmitoyl phosphatidylcholine (DPPC)/cholesterol mixed monolayer in the presence of high sodium ion levels was studied when the proportion of cholesterol was 50%. The results show that the effect of AmB on reducing the monolayer’s area at a high level of sodium ions is slightly stronger at 30 mN/m. The effect of AmB on reducing the elastic modulus of the DPPC/Chol monolayer is significantly weakened by a high sodium ion level, compared with the level of sodium ions at normal physiological concentration. The higher the sodium ion concentration, the weaker the intermolecular force of the DPPC/Chol/AmB mixed monolayers. The scanning electron microscope (SEM) and atomic force microscopy (AFM) observations suggest that at a high sodium ion level, the presence of AmB significantly reduces the surface roughness of the DPPC/Chol monolayer. AmB may bind to cholesterol molecules, and it isolates cholesterol from the monolayer, resulting in a reduced height of the cholesterol-rich monolayer and an increasingly dispersed monolayer region. The results are beneficial to understanding the mechanism of impact of a high sodium ion level on the relationship between AmB and red blood cell membranes rich in cholesterol and are valuable for understanding the hemolytic toxicity of AmB to red blood cells at a high sodium ion level.

## 1 Introduction

Invasive fungal infections carry a high mortality rate (Hagiya et al., 2023). In particular, critically ill patients, such as intensive care unit (ICU) patients, advanced cancer patients, or severe burn patients, are prone to fungal infection due to low immunity, which leads to disease aggravation or death (Smith, 2010; Candel et al., 2020; Matthew et al., 2020; Wen et al., 2022). Among the existing antifungal drugs, amphotericin B (AmB) has the broadest spectrum of activity and is the gold standard for the clinical treatment of invasive fungal infections (Kumari et al., 2022). Due to the limitation of drug safety indicators, AmB cannot be used in clinically effective doses due to kidney injury or hematotoxicity, which greatly affects the efficacy (Kotani et al., 2013). The key reason for the toxicity of this drug is its high affinity with cholesterol, which damages the cell membrane structure (Fournier et al., 2008). The affinity is due to a consequence of hydrogen bond formation between the OH group of cholesterol and the protonated amino group of AmB (Dynarowicz-Lątka et al., 2002). It is possible that two horizontally oriented AmB molecules and one dipalmitoyl phosphatidylcholine (DPPC) molecule in the vertical position form a stable complex that affects the thermodynamic properties of phospholipid membrane (Minones et al., 2004). At the same time, in recent years, some studies have found that AmB has the potential to induce immunogenic cell death and that induction is relative to the effect of AmB on the cell membrane (Medoff et al., 1974; Stein et al., 1982). With the gradual discovery of more functions of AmB, it is increasingly urgent to investigate the interaction mechanism between AmB and phospholipids in cell membranes. In addition to systemic fungal infections, increased mortality in severely ill patients is also associated with abnormal serum sodium concentration levels, especially in patients with severe burns (Sen et al., 2017; Grim et al., 2021). Many patients have abnormal blood sodium levels during ICU admission, and in patients with hypernatremia (serum sodium concentration greater than 145 mmol/L), the mortality rate was relatively increased (Oude et al., 2016). When patients are in this complex clinical environment, the effect of dose-limited amphotericin B in the treatment of antifungal infection may be affected. It is of practical significance to study the interaction between AmB and phospholipids in cell membranes at the condition of a high sodium ion level.

Studies have shown that the relationship between AmB and the cell membrane can be affected by the metal cation (Gagoś and Arczewska, 2011; Becucci et al., 2013). According to one report, a high sodium intake (>4 mEQ/kg) may inhibit the toxicity of AmB to the kidneys (Turcu et al., 2009). It is also proposed that AmB may hinder the ATP (Na^+^-K^+^) activity in animal cells (Brajtburg and Bolard, 1996; Arczewska and Gagoś, 2011). The impact of potassium ions on the interaction between AmB and membrane has been investigated in our previous works. Although sodium ions and potassium ions are both monovalent ions, their impacts on the interaction between AmB and phospholipid membranes are different due to their different sizes (Arczewska and Gagoś, 2011). The impact of sodium ions on the relationship between AmB and the phospholipid membrane or the phospholipid-cholesterol membrane has been studied using a vesicle bilayer as a cell membrane model (Wang et al., 2017). When cholesterol is present in the membrane system, the impact of sodium ions on the membrane system is clearly different than the impact on systems without cholesterol. The Na^+^ ions can promote cholesterol to enhance the fluidity of the phospholipid membrane, which may be the main reason why sodium ions affect the interaction between AmB and the membrane.

Cholesterol is an important sterol on the cell membrane and is also a lipid that forms a functional lipid raft (Morgan et al., 2020). Among all the lipids in the cell membrane, the proportion of cholesterol molecules reaches approximately 30 mol% (Nigam, 2020). The interaction between AmB and phospholipid membrane has been studied at this cholesterol ratio (Wang et al., 2020a; Wang et al., 2020b). However, this drug faces a complex cellular environment after entering the body, and when administered intravenously, it comes into contact with red blood cells. The blood toxicity of AmB is dependent on the destruction of the erythrocyte membrane by AmB. In red blood cells, the proportion of cholesterol molecules in membrane lipids is as high as 50 mol% (Rosenhouse-Dantsker et al., 2023). Cholesterol is a regulator of signaling molecules and functional proteins, and it also happens to be the binding site of AmB affinity to cell membranes. Dipalmitoyl phosphatidylcholine (DPPC) is a kind of saturated phospholipid that is often present in the outer cell membrane leaflet (Wnętrzak et al., 2021). Due to its clear phase transitions and well-defined mechanical and thermodynamic properties, DPPC has been widely used in Langmuir monolayers as a membrane model (Ruiz et al., 2020). A mixed membrane composed of DPPC and cholesterol has been widely employed to research the drug/protein-lipid interaction (Tovani et al., 2013; Wang et al., 2020a; Pereira et al., 2021). The interaction between amphotericin B and the phospholipid membrane where much cholesterol accumulates is noteworthy, and it is helpful to understand the mechanism of hematotoxicity caused by AmB damage to the erythrocyte membrane.

The vesicle bilayer is a simplified model used to study the cell membrane system *in vitro* (Träger et al., 2023), and the curvature and size of the bilayer are close to the real cell membrane. However, the composition of the bilayer is difficult to control precisely, and information about the thermodynamic characteristics of the membrane system cannot be obtained. A Langmuir monolayer model can accurately control components of membrane, environment, temperature, and other conditions, and it is very suitable for exploring the lipid–drug interaction (Pires et al., 2019; Pires et al., 2020; Jurak et al., 2021; Jochelavicius et al., 2022). The surface pressure and the molecular packing of the lipid membrane can be modified in the monolayer model (Pivetta et al., 2023). The AmB molecule consists of a fungal amine group and a large lactone ring with seven double bonds that are connected to the main ring by glycosidic bonds that have a very rigid structure. It is an amphiphilic molecule with both hydrophobic (polyolefin chain) and hydrophilic (polyhydroxyl chain) properties (Oliveira et al., 2021). This is very suitable for the Langmuir monolayer model to analyze the interaction between AmB and biomembrane at high sodium ion levels.

Therefore, in this work, the association between AmB and a saturated phospholipid monolayer containing 50 mol% cholesterol molecules in the presence of Na^+^ ions has been explored by the Langmuir method, scanning electron microscopy, and atomic force microscopy. Moreover, the findings are conducive to comprehending the mechanism of the influence of high sodium ion levels on the interaction between drugs and lipid membranes rich in cholesterol when AmB interacts with the outer membrane of red blood cells.

## 2 Materials and methods

### 2.1 Materials

All powder lipids and drugs were purchased from Sigma, United States. The purity of lipids is found to be greater than 99%. The purity of the purchased amphotericin B was greater than 80%; it was purified to 99% by means of HPLC mentioned in the literature (Arczewska and Gagoś, 2011) before use. Before the experiment, the lipid mixture and the drug solution were prepared by dissolving a 1:1 M ratio of phospholipids and cholesterol into a chloroform/methanol mixture (9:1, v/v) to form a 0.7 mM mixed solution. Amphotericin B was dissolved into a 3:1 (v/v) mixture of dimethylformamide and 1 M HCl to form a drug solution with a concentration of 0.7 mM. The aqueous solution at the air-water interface was a 20 mM HEPES (N-2-hydroxyethylpiperazine-N-2-ethanesulfonic acid) buffer with a stable pH value of 7.0. Sodium ions were dissolved into a HEPES buffer to form an interface environment with different concentrations (0 mM, 140 mM, and 280 mM) of sodium ions. The ultrapure water applied in the experiment came from a Milli-Q plus water purification system (18.2 MΩ/cm, Millipore, United States).

### 2.2 Langmuir technique

An air–water interface was offered by the Langmuir trough (KSV-Minitrough, Finland). The monolayer at the interface can be compressed inward or expanded outward by two Teflon barriers at a certain speed. In this process, a Wilhelmy-type tensiometer was adopted to detect the change in the monolayer’s surface pressure.

First, before conducting the experiment, the Teflon trough and two Teflon barriers were cleaned with ethanol to ensure that no other impurities were mixed. Second, 200 mL HEPES with or without Na^+^ ions were added to the trough. Third, a Hamilton microsyringe was used to extract 20 μL of the mixed lipid solution, which was dripped onto the surface of the HEPES buffer. For the AmB-lipid monolayer experiment, add 10 μL lipid mixture and 10 μL drug solution onto the surface of the buffer successively. The solvent was allowed to evaporate for 10 min. The monolayer was compressed by 7 mm/min, and the surface pressure-mean molecular area isotherms ( π−A ) of the monolayer were measured. When the monolayer was compressed to 30 mN/m, the pressure was stopped and kept constant, and the surface pressure change over time was measured. All experiments were repeated three times to ensure the repeatability of the data. An external circulator was used to keep the temperature at 35 ± 1 °C.

### 2.3 Scanning electron microscope and atomic force microscopy

After the monolayer was compressed to a fixed surface pressure, the surface pressure was kept constant. The Langmuir monolayer was transferred with a dipping rate of 1 mm/min onto the fresh mica at 30 mN/m to form the Langmuir–Blodgett (LB) film (Nepal and Stine, 2023), the morphology of which can be observed through a scanning electron microscope (SEM, Hitachi, Japan) and atomic force microscopy (AFM, Shimadzu, Japan). The monolayer films were coated with gold before the SEM examination, and the SEM images were obtained in the range of 20 μm×20 μm . The film samples for AFM testing were not otherwise processed. The force constant of the probe used in AFM testing is 0.1 N/m. The morphology of monolayer films was observed in the intermittent contact mode.

## 3 Results and discussion

### 3.1 Surface pressure–mean molecular area isotherms

Compared with the three-dimensional system, the monolayer on the interface can be regarded as a two-dimensional system. In the process of compression, the π−A isotherm reflects the change process of different phase states of monolayer, including gaseous, liquid-expanded (LE), liquid-condensed (LC), solid, and intermediate or transition films (Tovani et al., 2013; Nepal and Stine, 2023). Three characteristic parameters are usually defined on the isotherm to analyze the phase behavior of monolayers (Chen et al., 2007; Wang et al., 2018): the liftoff area $A_{L}$ , the limiting area $A_{\infty }$ , and the collapse pressure $\pi_{C}$ . The mean molecular area is called the liftoff area $A_{L}$ when the π−A isotherm increases from the baseline. The limiting area $A_{\infty }$ can be calculated by extrapolating the slope of the isotherm in its steepest range to the zero-surface pressure (Devterova et al., 2020), which is an empirical parameter around the mean molecular cross-sectional area. At the high surface pressure of the isotherm, with increasing surface pressure, the mean molecular area suddenly decreases, indicating that the monolayer has collapsed. The surface pressure and mean molecular area at collapse are, respectively, the collapse pressure $\pi_{C}$ and collapse area $A_{C}$ .

From Table 1, for the DPPC monolayer containing 50 mol% cholesterol, the values of parameters $A_{L}$ , $A_{\infty }$ , and $\pi_{C}$ all gradually tend to increase with increasing sodium ion concentration in the subphase without amphotericin B. However, there is no significant pattern in the trend of the $A_{C}$ value with the growing sodium ion concentration. When the concentration of sodium ions is 140 mM, the $A_{C}$ value reached a minimum, which may be due to the collapse of the monolayer folds or the formation of multiple layers.

**TABLE 1:** *A*_*L*_, *A*_*∞*_, *π*_*C*_, and *A*_*C*_ parameter values of the DPPC monolayer that contains 50 mol% cholesterol with or without AmB in the presence of sodium ions.

Monolayer	Concentration of Na^+^/mM	$A_{L}/{Å}^{2}$	$A_{\infty }/{Å}^{2}$	$\pi_{C}/mN/m$	$A_{C}/{Å}^{2}$
DPPC/Chol (1:1)	0	59.58 ± 0.31	50.72 ± 0.22	52.80 ± 0.11	32.38 ± 0.29
	140	72.92 ± 0.21	52.71 ± 0.27	54.15 ± 0.10	29.31 ± 0.24
	280	88.75 ± 0.29	61.14 ± 0.23	60.48 ± 0.12	33.04 ± 0.27
DPPC/Chol/AmB (0.5:0.5:1)	0	125.41 ± 0.28	48.74 ± 0.23	34.47 ± 0.11	23.34± 0.36
	140	113.08 ± 0.35	40.07 ± 0.34	42.79 ± 0.13	23.29 ± 0.37
	280	125.11 ± 0.19	48.34 ± 0.31	47.19 ± 0.15	23.13 ± 0.31

When amphotericin B was added to the monolayer, the value of $\pi_{C}$ gradually increases with the increasing sodium ion concentration, and the $A_{C}$ value at collapse gradually decreases. Compared to the absence of sodium ions, the values of $A_{L}$ and $A_{\infty }$ of the lipid/AmB mixed monolayer decrease at a concentration of 140 mM sodium ions, while the values of $A_{L}$ and $A_{\infty }$ do not change significantly in the presence of 280 mM sodium ions.

**FIGURE 1:**
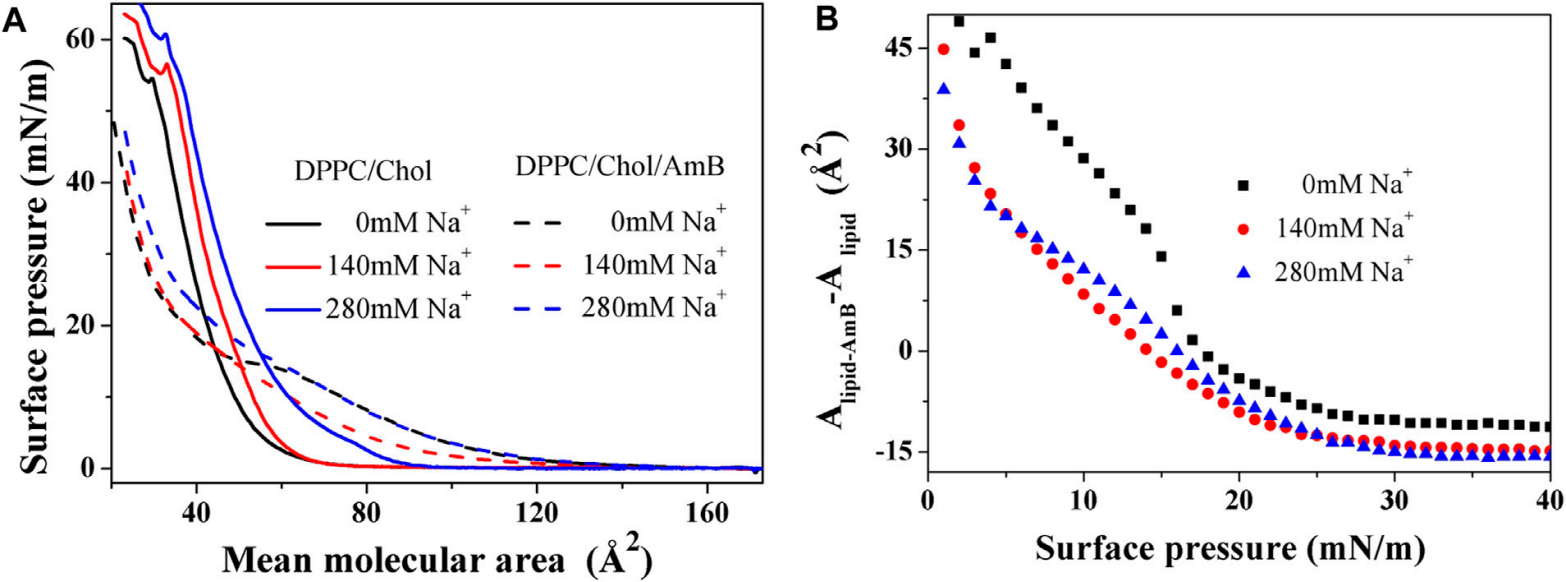
π−A isotherm of a DPPC monolayer containing 50 mol% cholesterol with or without AmB in the presence of sodium ions **(A).** The increase in the mean molecular area caused by AmB varies with the surface pressure **(B)**.

From Figure 1B, when the surface pressure is lower than 15 mN/m, the increment of monolayer area induced by AmB is reduced by sodium ions under the same surface pressure. When the surface pressure is higher than 17 mN/m, AmB reduces the monolayer area, and sodium ions can enhance this effect. When the surface pressure is greater than 28 mN/m, the reduction of monolayer area caused by AmB tends to be stable, and the effect of AmB at the high level of sodium ions is slightly stronger than that at 140 mM concentration of sodium ions.

### 3.2 The modulus of elasticity for the monolayers

The compressibility of monolayers is often characterized by an important parameter, the modulus of elasticity, obtained by the data of a π−A isotherm using the following formula (Davies and Rideal, 1963; Krajewska et al., 2013; Machado and Caseli, 2020):

$$C_{s}^{-1}=-A{\left(\frac{\partial \pi }{\partial A}\right)}_{T},$$ (1)

where *s* refers to the cross-sectional area of the monolayer at the interface, *A* indicates the mean molecular area, and *π* denotes the surface pressure. A large $C_{s}^{-1}$ value suggests low compressibility of the monolayer.

**FIGURE 2:**
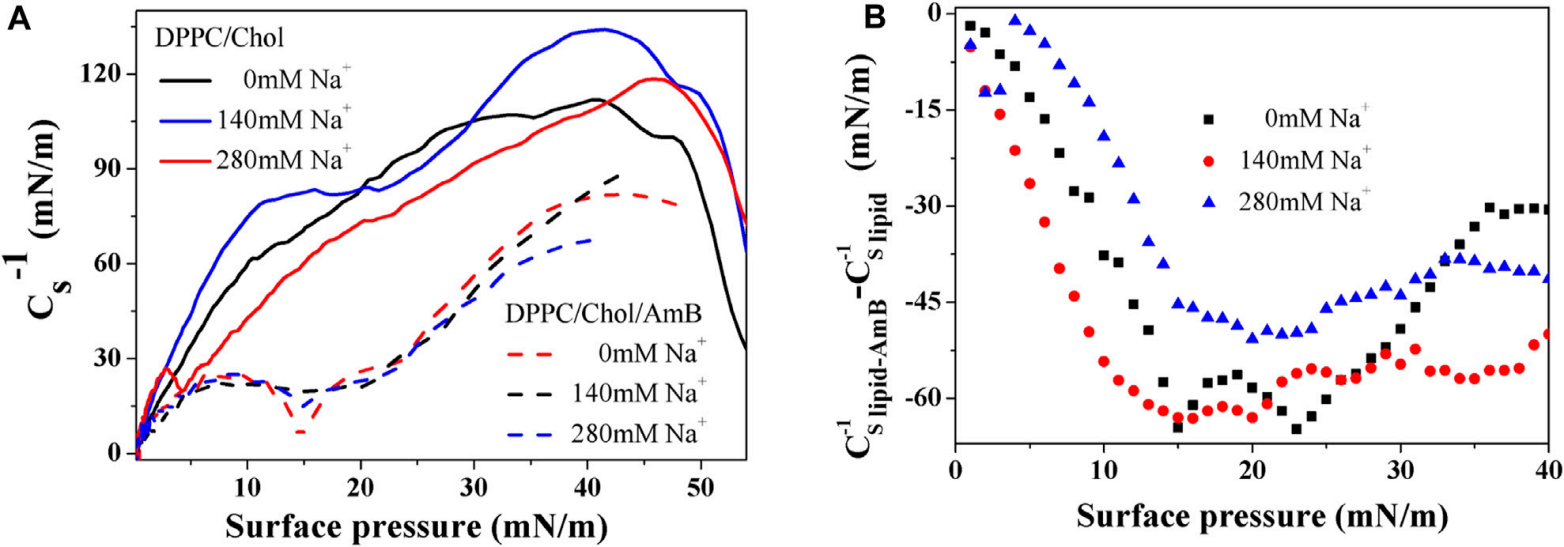
$C_{s}^{-1}-\pi$ curves of DPPC monolayer containing 50 mol% cholesterol with or without AmB in the presence of sodium ions **(A).** The increase of $C_{s}^{-1}$ caused by AmB varies with the surface pressure **(B)**.

Based on Figures 2A,B, the elastic modulus of the DPPC monolayer, including 50 mol% cholesterol, gradually decreases with the increasing sodium ion concentration in the 5–40 mN/m range of surface pressures, suggesting the compressibility and disorder of the lipid monolayer increases. When amphotericin B is present, the $C_{s}^{-1}$ value of mixed monolayers all decreases compared with the DPPC monolayer containing 50 mol% cholesterol. This may be due to the formation of the compound of AmB and cholesterol (Borzyszkowska-Bukowska et al., 2023). The presence of sodium ions significantly causes the $C_{s}^{-1}$ value to decrease in the 18.7–30 mN/m range of surface pressures. Nevertheless, the $C_{s}^{-1}$ values are little different in the presence of 140 mM and 280 mM sodium ions. At the same time, there is a minimum value for the elastic modulus of the DPPC/Chol/AmB mixed monolayer in the absence of sodium ions, consistent with a phase transition. The reason for this is that the orientation of the AmB molecule is altered to a vertical direction from a horizontal direction (Arczewska and Gagoś, 2011). The degree of influence of AmB on the elastic modulus of the DPPC/Chol monolayer is interfered with by the sodium ion levels (Figure 2B). Compared with the level of sodium ions at normal physiological concentration, the effect of AmB on reducing the elastic modulus of DPPC/Chol monolayer is significantly weakened by high sodium ion level.

### 3.3 Intermolecular interaction from excess Gibbs free energy

The excess Gibbs free energy of mixing ${∆G}^{exc}$ can indicate the interaction force between the molecules on the monolayer. It was calculated for the Langmuir monolayer composed of three components at a given π value according to the following formula (Gzyl-Malcher et al., 2012):

$$∆G^{exc}=N\int_{0}^{\pi }A^{exc}d\pi ,$$ (2)

where N refers to Avogadro’s number and $A^{exc}$ represents the excess area defined as follows (Panda et al., 2009):

$$A^{exc}=A_{123}-A_{1}X_{1}-A_{2}X_{2}-A_{3}X_{3},$$ (3)

where *A*_*123*_ is the mean molecular area of the mixed monolayer, and *A*_*1*_, *A*_*2*_, and *A*_*3*_ are the mean molecular areas of the pure monolayer of components 1, 2, and 3, respectively, at a given π value. *X*_*1*_ *, X*_*2*_, and *X*_*3*_ are, respectively, the molar fractions of components 1, 2, and 3 in the mixed monolayer. A negative value of ${∆G}^{exc}$ suggests an attractive force of intermolecular interaction (Marsh, 1996). The absolute value of ${∆G}^{exc}$ is large, meaning the force is strong.

**FIGURE 3:**
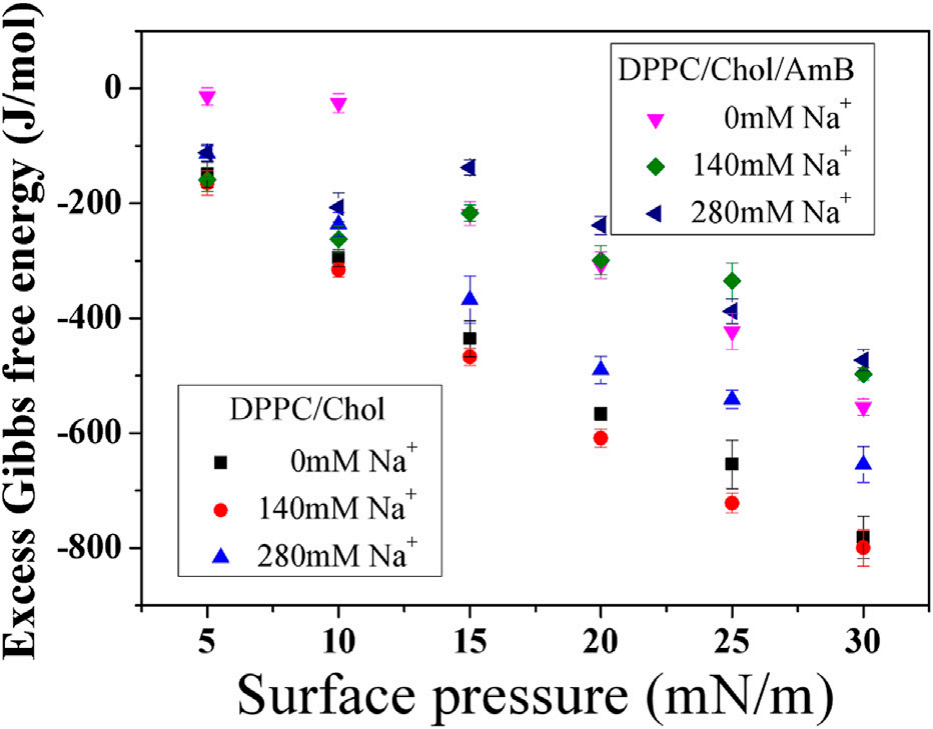
Excess Gibbs free energy of mixing ( ${∆G}^{exc}$ ) of DPPC monolayers containing 50 mol% cholesterol with or without AmB in the presence of sodium ions at different surface pressures.

According to Figure 3, the ${∆G}^{exc}$ value of the DPPC/Chol mixed monolayer is negative, which is consistent with the literature (Chou and Chang, 2000; Gong et al., 2002). The value is negative in all cases of the lipid–drug mixed monolayer, suggesting that the intermolecular force shows up as attraction. The absolute value of $∆G^{exc}$ of the DPPC/Chol/AmB system is all less than that of DPPC/Chol system, regardless of the presence or concentration of sodium ions. The findings indicate that the intermolecular force of the lipid–drug mixed system is smaller than that of the lipid system. The intermolecular force between DPPC and cholesterol is weakened due to the formation of the AmB-Chol complex. It is suggested that the incorporation of the AmB drug is thermodynamically unfavorable and induces membrane destabilization, which is consistent with the literature (Hąc-Wydro et al., 2009). In order to make the analysis results of the monolayer more biologically significant, the results at 30 mN/m were discussed. At 30 mN/m, the properties of pressure, area per molecule, phase transition, and compressibility of monolayer films are similar to those of natural bilayer films (Marsh, 1996). At 30 mN/m, the intermolecular force of the DPPC monolayer containing 50 mol% cholesterol is insensitive to the sodium ions in low concentration. With a high concentration of sodium ions, the intermolecular force is obviously weakened. Differently, the intermolecular force of the DPPC/Chol/AmB mixed monolayers shows a clear rule with the change of sodium ion concentration: the higher the sodium ion concentration, the weaker the intermolecular force.

### 3.4 Relaxation of the DPPC monolayers containing 50 mol% cholesterol with or without AmB at constant area

**FIGURE 4:**
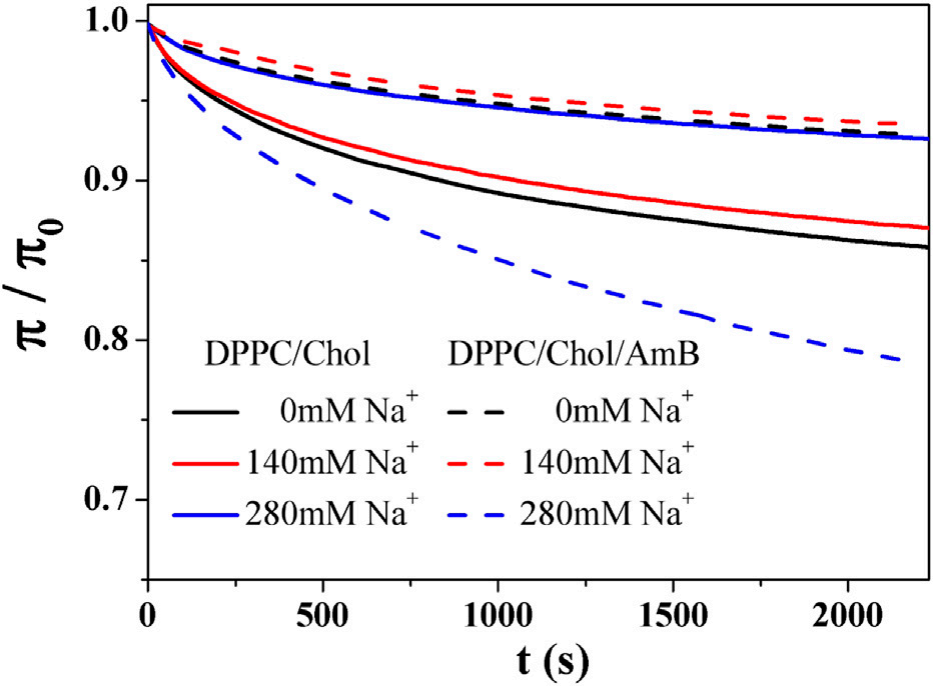
$\pi /\pi_{0}-t$ curves of DPPC monolayers containing 50 mol% cholesterol with or without AmB in the presence of Na^+^ ions.

The stability of the mixed monolayers was studied by a relaxation experiment at an initial surface pressure ( $\pi_{0}$ ) of 30 mN/m. The relaxation process of monolayers was indicated by the π−t curves at constant area (Robinson et al., 2020). The π−t curves are normalized to get the $\pi /\pi_{0}-t$ curves (Figure 4). Some characteristic parameters (Table 2) of relaxation can be acquired through fitting $\pi /\pi_{0}-t$ curves with the following formula (Chen et al., 2002; Wang et al., 2018):

$$\pi /\pi_{0}=C+ae^{-t/\tau },$$ (4)

where C represents the normalized equilibrium pressure, and τ indicates the lifetime associated with the reorganization of monolayers. The large τ value denotes that the monolayer is disordered, and the amount of time before the conformation transition of the monolayer becomes longer (Wang et al., 2018).

**TABLE 2:** Values of $C,a,\tau ,\text{and }r^{2}$ obtained by fitting $\pi /\pi_{0}-t$ curves.

Monolayer	Concentration of Na^+^/mM	C	a	τ	$r^{2}$
DPPC/Chol (1:1)	0	0.850	0.130	827.458	0.992
	140	0.864	0.117	867.175	0.992
	280	0.920	0.069	986.793	0.990
DPPC/Chol/AmB (0.5:0.5:1)	0	0.922	0.069	1,014.246	0.990
	140	0.926	0.068	1,100.065	0.992
	280	0.753	0.221	1,200.474	0.996

Table 2 shows that the C value of DPPC/Chol mixed monolayer increases as the concentration of sodium ions increases, suggesting the monolayer is more stable at higher concentrations of sodium ions. However, for the DPPC/Chol/AmB mixed monolayer, the C value decreases at 280 mM Na^+^ ions, meaning sodium ions interfere with the stability of AmB/lipid monolayer film when sodium ions are in a high concentration. When the concentration of sodium is constant, the τ value of the saturated phospholipid/AmB mixed monolayer is greater than that of the saturated phospholipid monolayer containing 50 mol% cholesterol, indicating that the presence of AmB increases the disorder of the saturated phospholipid monolayer containing 50 mol% cholesterol. This is consistent with the result that AmB reduces the $C_{s}^{-1}$ value of the saturated phospholipid monolayer containing 50 mol% cholesterol. The τ value of the DPPC monolayers containing 50 mol% cholesterol varies with the concentration of sodium ions and is not affected by AmB. The τ value is larger at the higher concentration of sodium ions, indicating the disorder of the stronger ternary mixed monolayer film.

### 3.5 Morphology of mixed monolayer by SEM and AFM

**FIGURE 5:**
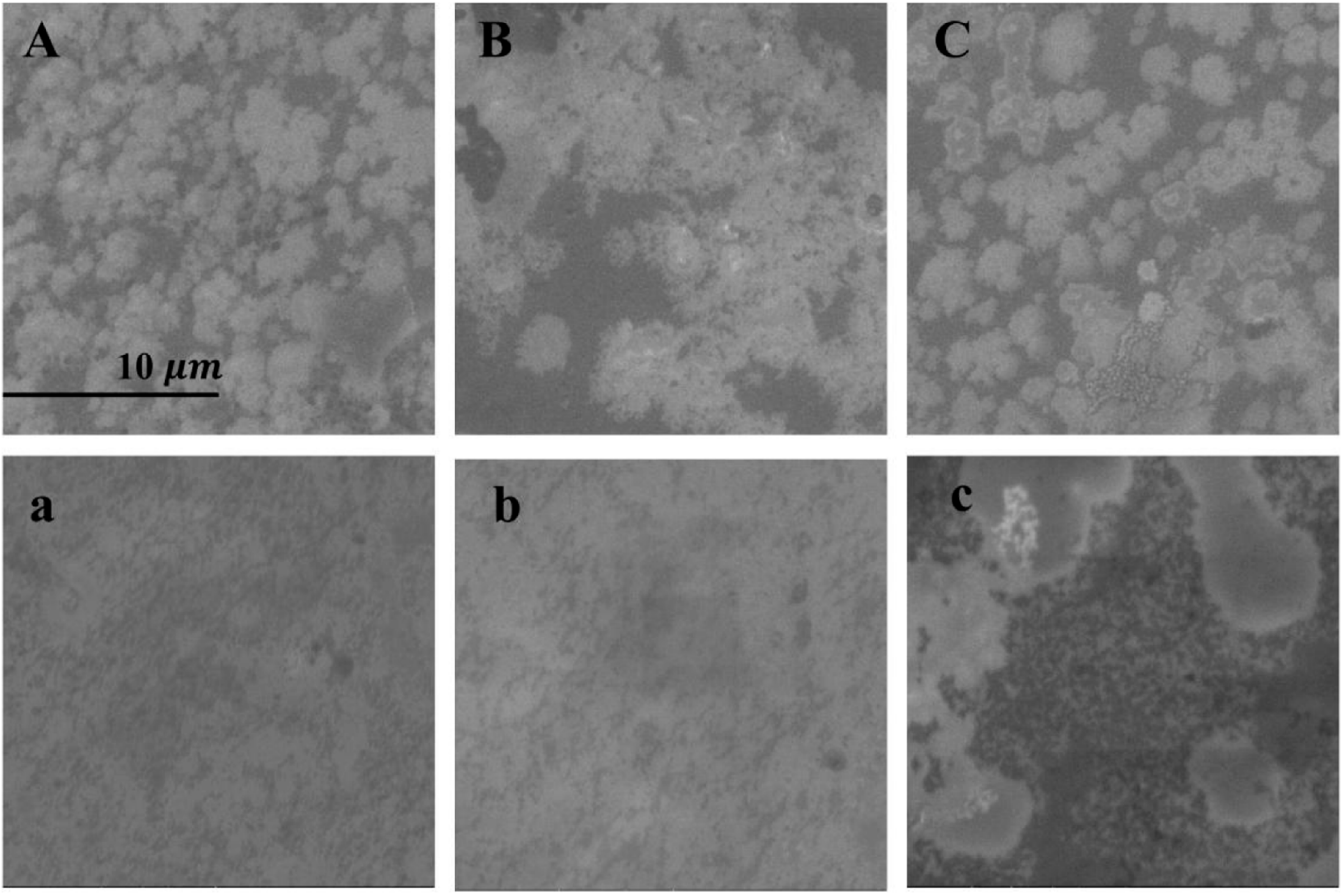
SEM images ( 20 μm×20 μm ) of the DPPC/Chol monolayer film **(A–C)** and the DPPC/Chol/AmB monolayer film **(a, b, c)** in the presence of 0 mM **(A, a)**, 140 mM **(B, b),** and 280 mM **(C, c)** Na^+^ ions at 30 mN/m.

The DPPC/Chol (1:1) monolayer and the DPPC/Chol/AmB (0.5:0.5:1) monolayer at the air–water interface were separately transferred onto a mica sheet to form the LB films. The surface morphology of these films was obtained by SEM and AFM. In Figure 5, without sodium ions, the bright regions of the DPPC/Chol monolayer film are flaky with clear edges. When the concentration of sodium ions is 140 mM, the flaky edges of the bright regions are fuzzy and show subtle burrs. The monolayer film has obvious spot-like micro areas in the presence of 280 mM Na^+^ ions. When AmB is present in the DPPC/Chol monolayer, the mixed film appears lamellar in the absence of Na^+^ ions, but unlike the DPPC/Chol film, the DPPC/Chol/AmB mixed film looks more irregular and has unclear boundaries. With 140 mM Na^+^ ions, the morphology of DPPC/Chol/AmB monolayer films is similar to that in the absence of sodium ions. However, with 280 mM Na^+^ ions, large sheets with clear boundaries and many small and dispersed regions are observed in the DPPC/Chol/AmB monolayer films.

**FIGURE 6:**
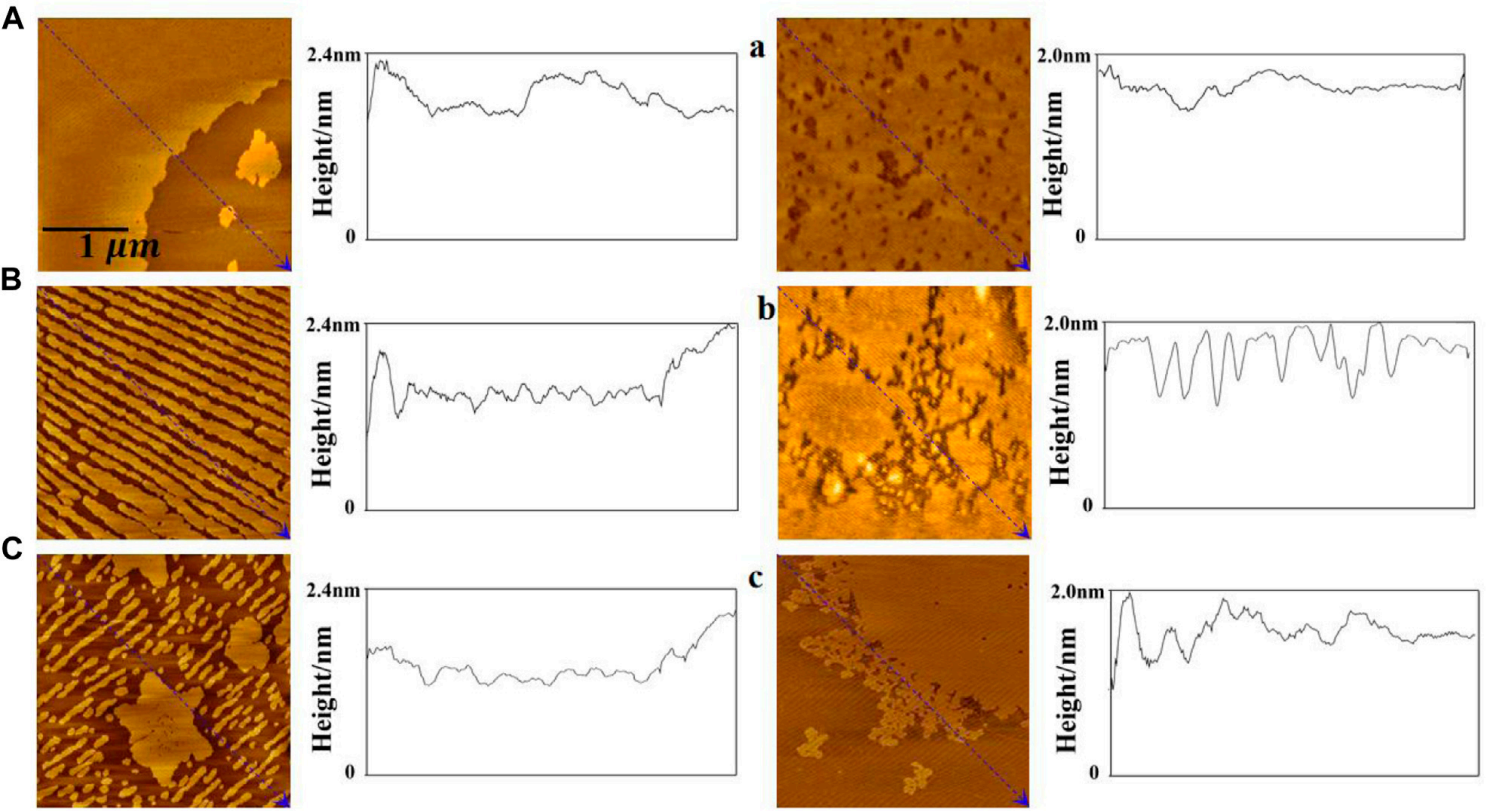
AFM images of the DPPC/Chol monolayers **(A–C)** and the DPPC/Chol/AmB monolayers **(a, b, c)** in the presence of 0 mM **(A, a)**, 140 mM **(B, b),** and 280 mM **(C, c)** Na^+^ ions at 30 mN/m.

The atomic force microscopy images show the morphological details of the monolayer. From Figure 6, the height of the bright region of the DPPC/Chol monolayer is 1.9 nm–2.4 nm, which is similar to the literature (Wang et al., 2023), and it is almost unaffected by sodium ions. In the absence of sodium ions, the bright region of the DPPC/Chol monolayer film is a large flake, which is dispersed into small strip regions in the presence of 140 mM Na^+^ ions. In the presence of 280 mM Na^+^ ions, irregular flake regions and numerous smaller strip regions are found on the DPPC/Chol monolayer film. The light region of the DPPC/Chol/AmB monolayer film in the absence of sodium ions is also flaky, but there are scattered dark regions on the film. In the presence of 140 mM sodium ions, the dark regions are enlarged and connected, and the lamellar regions of the film are separated by dark divisions. In the presence of 280 mM Na^+^ ions, the dark regions are larger, and small scattered regions are observed at the edge of the lamellar region. At the same time, in the absence of sodium ions, the height of the bright regions on the DPPC/Chol/AmB monolayer decreases to 1.7 nm–2.0 nm due to the presence of AmB, which is consistent with the literature (Chou and Chang, 2000). Similar to that of the DPPC/Chol monolayer film, the height of bright regions on the DPPC/Chol/AmB monolayer is also unaffected by sodium ions. The results of AFM image analysis and SEM image analysis confirm each other. The results of the AFM and SEM image analyses show that the change of the morphology caused by AmB and sodium ions is basically consistent with the change rule of monolayer disorder mentioned in the results of elastic modulus analysis.

The surface roughness of the mixed monolayer film is obtained from AFM images and reported in Table 3. In the absence of AmB, the roughness of DPPC/Chol monolayer film gradually increases with the increasing sodium ion concentration, which is different from its roughness in the presence of AmB. At the normal physiological concentration of sodium ions, the effect of AmB on the surface roughness of the DPPC/Chol monolayer is not significant. At the high sodium ion level, the presence of AmB significantly reduces the surface roughness of the DPPC/Chol monolayer.

**TABLE 3:** Root mean square roughness (RMS-R_q_) and roughness average (R_a_) in monolayer film AFM images.

Monolayer	Concentration of Na^+^/mM	RMS-R_q_ (nm)	R_a_ (nm)
DPPC/Chol	0	0.201 ± 0.002	0.152 ± 0.008
	140	0.593 ± 0.009	0.412 ± 0.011
	280	0.715 ± 0.010	0.524 ± 0.010
DPPC/Chol/AmB	0	0.256 ± 0.006	0.185 ± 0.007
	140	0.593 ± 0.008	0.425 ± 0.010
	280	0.296 ± 0.010	0.211 ± 0.010

**FIGURE 7:**
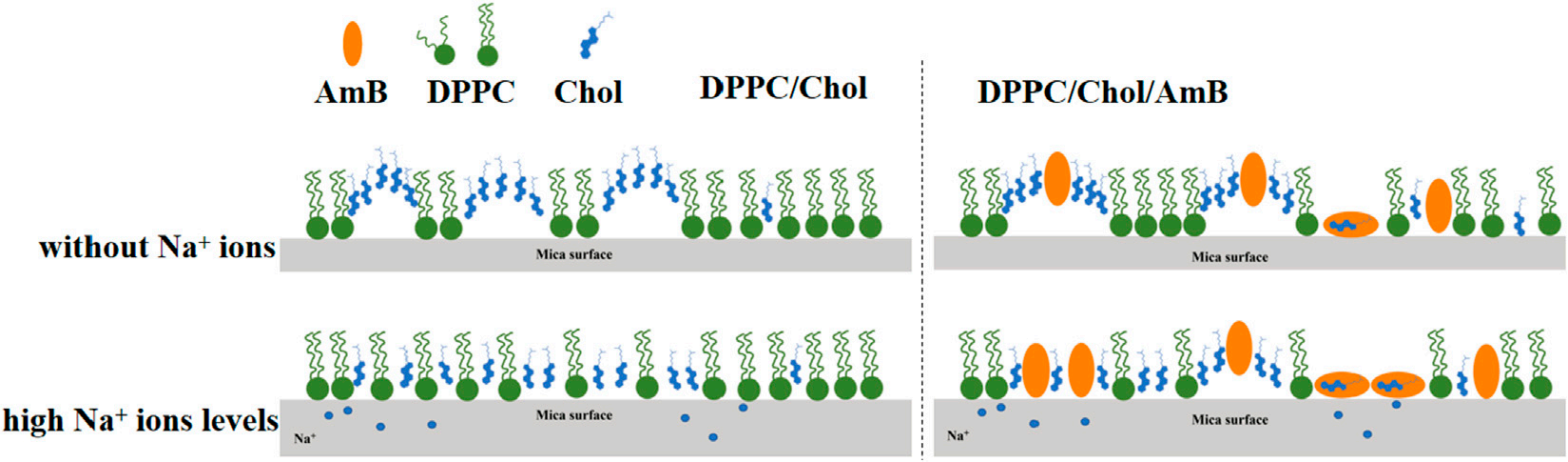
Molecular mechanism diagram for the impact of sodium ions on the interaction of AmB with a cholesterol-rich phospholipid membrane.

Amphotericin B binds to cholesterol molecules on cholesterol-rich phospholipid membranes (Figure 7), which may be mainly in three forms. 1) Amphotericin B binds to cholesterol accumulation areas; 2) After amphotericin B binds to cholesterol molecules, it is isolated from the aggregation area to form “ion channels,” and the long axes of amphotericin B molecules are perpendicular to the interface; 3) After amphotericin B binds to cholesterol molecules, the long axis is parallel to the interface, and the cholesterol is isolated from the phospholipid membrane like a “sponge.” The isolation effect of AmB on cholesterol is consistent with the “ion channels” model and “sponge” model theory (Cohen, 2010; Kamiński, 2014). AmB can weaken the intermolecular force of the phospholipid membrane and increase the disorder of monolayer, and sodium ions can make this effect more intense, which is closely related to the isolation of cholesterol by AmB. The presence of sodium ions in the ambient solution inhibits the accumulation of cholesterol in the DPPC/Chol monolayer. When amphotericin B interacts with a cholesterol-rich phospholipid membrane, a high concentration of sodium ions may promote the sequestration effect of AmB on cholesterol.

## 4 Conclusion

To conclude, the association between AmB and a cholesterol-rich phospholipid membrane was studied using the Langmuir monolayer method, scanning electron microscopy, and atomic force microscopy. According to thermodynamic analysis, at 30 mN/m the elastic modulus of the DPPC/Chol monolayer decreases due to sodium ions, and the greater the concentration of sodium ions, the greater the influence. Compared with the level of sodium ions at normal physiological concentration, the effect of AmB on reducing the elastic modulus of the DPPC/Chol monolayer is significantly weakened by high sodium ion levels. The high concentration of sodium ions weakens the intermolecular force of DPPC/Chol monolayers. When AmB is present, a low concentration of sodium ions could also significantly weaken the intermolecular attraction of the mixed monolayers. The relaxation results show that AmB tends to increase the disorder of the monolayer, and the effect is stronger with the increasing sodium ion concentration. The results of the SEM and AFM observations are in agreement with those of elastic modulus and relaxation analysis. Although the Langmuir monolayer model used in this experiment is different from the actual cell membrane in terms of membrane curvature, the composition and proportions of the monolayer can be accurately controlled. From the perspective of quantitative simulation of phospholipid membrane, the Langmuir monolayer model is well used to study the correlation between the cholesterol-rich membrane and drugs in the actual cell membrane. When the monolayer is compressed to 30 mN/m, the pressure, area per molecule, and phase transition of the monolayer are close to the natural bilayer membrane. AmB can form a complex with cholesterol (Kamiński, 2014), which changes the permeability of the cell membrane and ion channels formed in the phospholipid membrane. Important elements could escape from the cell, ultimately leading to cell death and causing hemolytic effects on red blood cells. The abnormal increase in the sodium ion concentration enhances the destruction of membrane stability by AmB, which may enhance the hemolysis effects of AmB drugs. The results of this study have some theoretical reference value for evaluating the potential toxicity of AmB in severely ill patients with fungal infections complicated with hypernatremia.

## Data Availability

The original contributions presented in the study are included in the article/Supplementary Material; further inquiries can be directed to the corresponding author.
